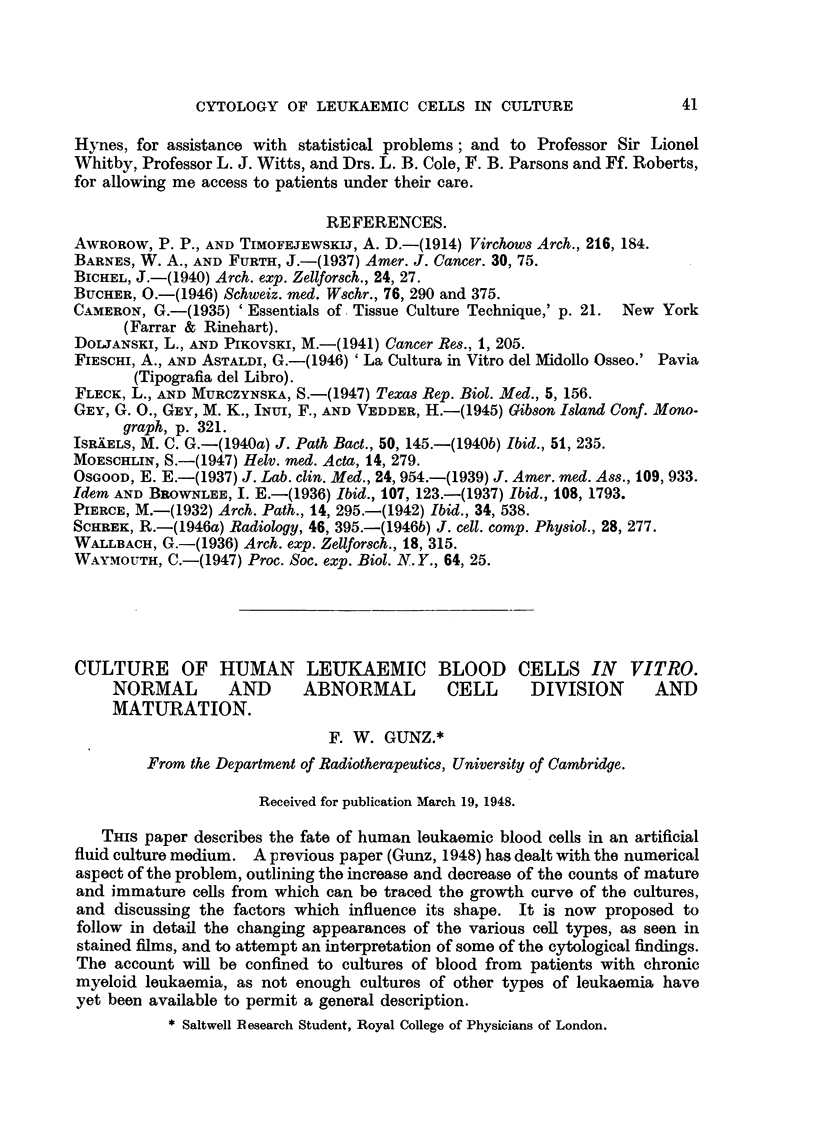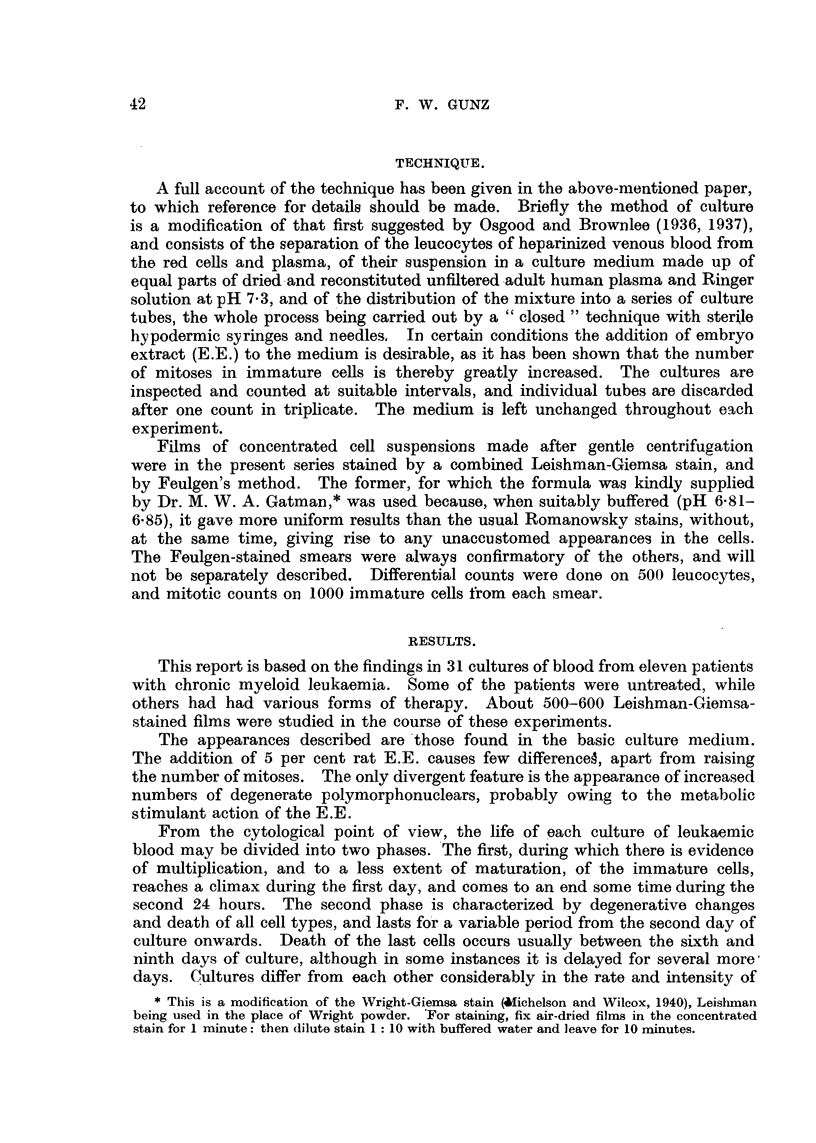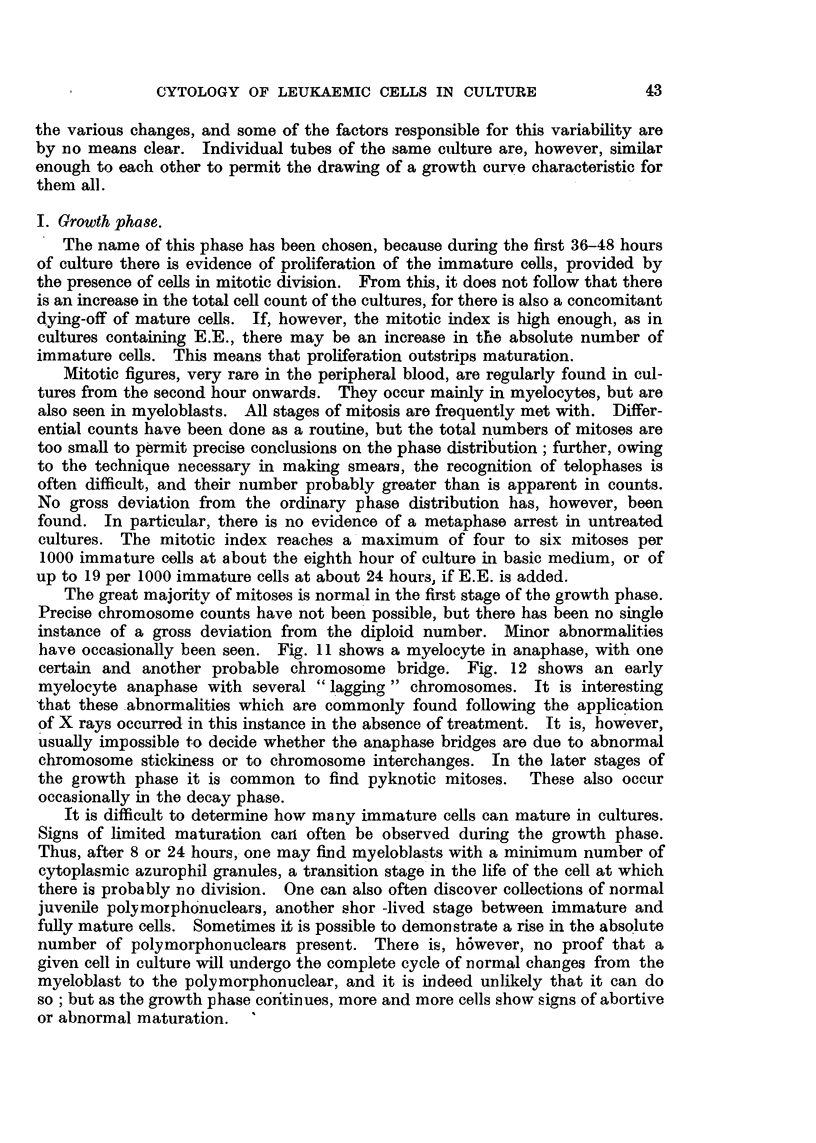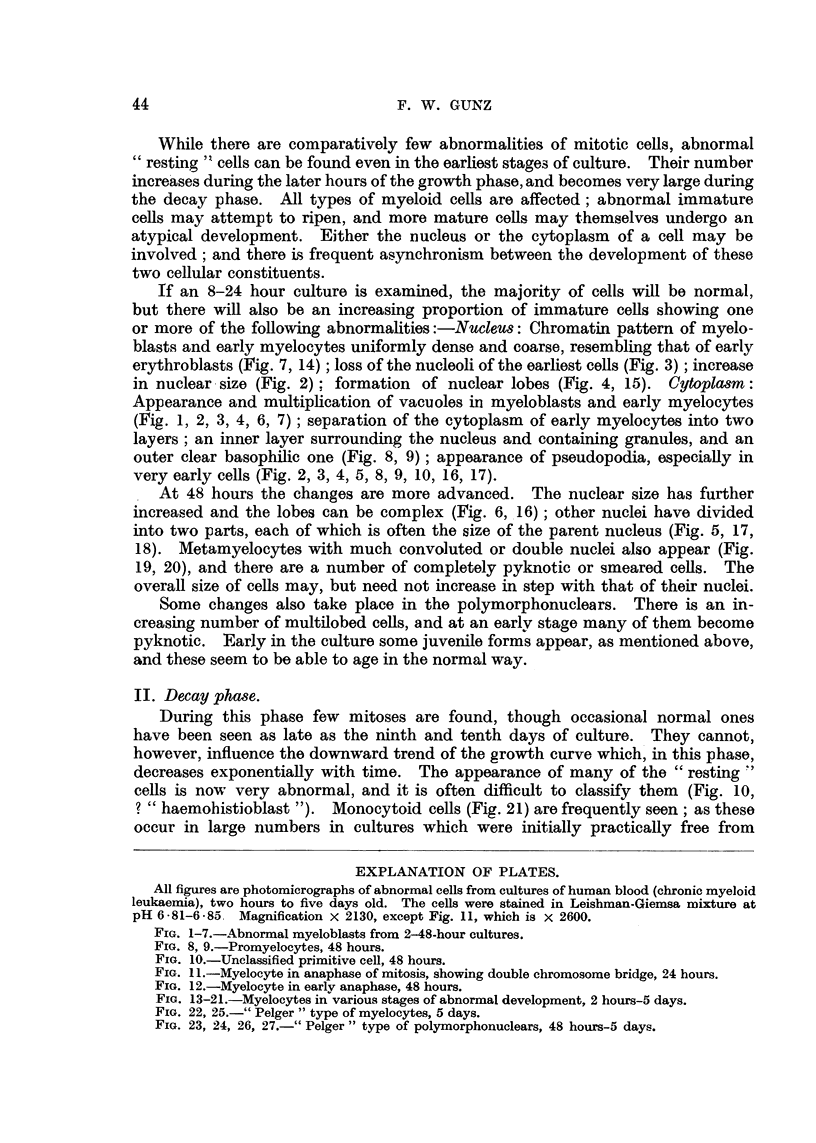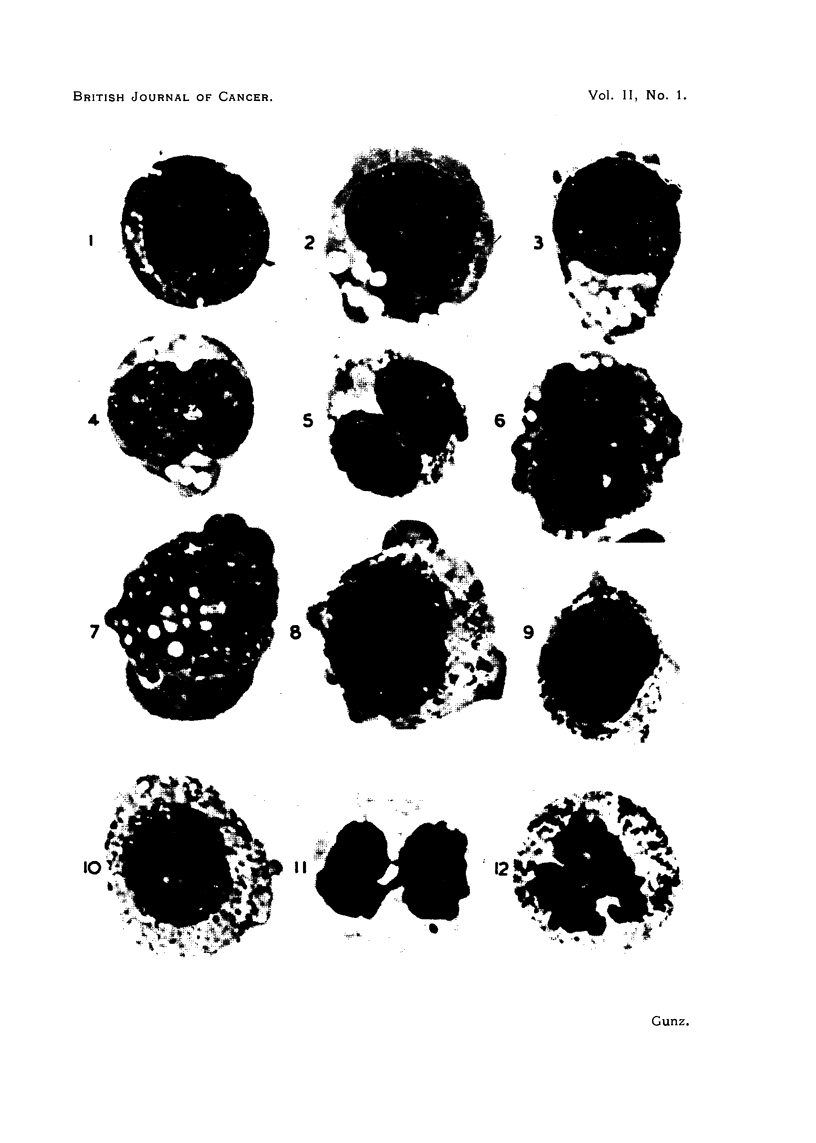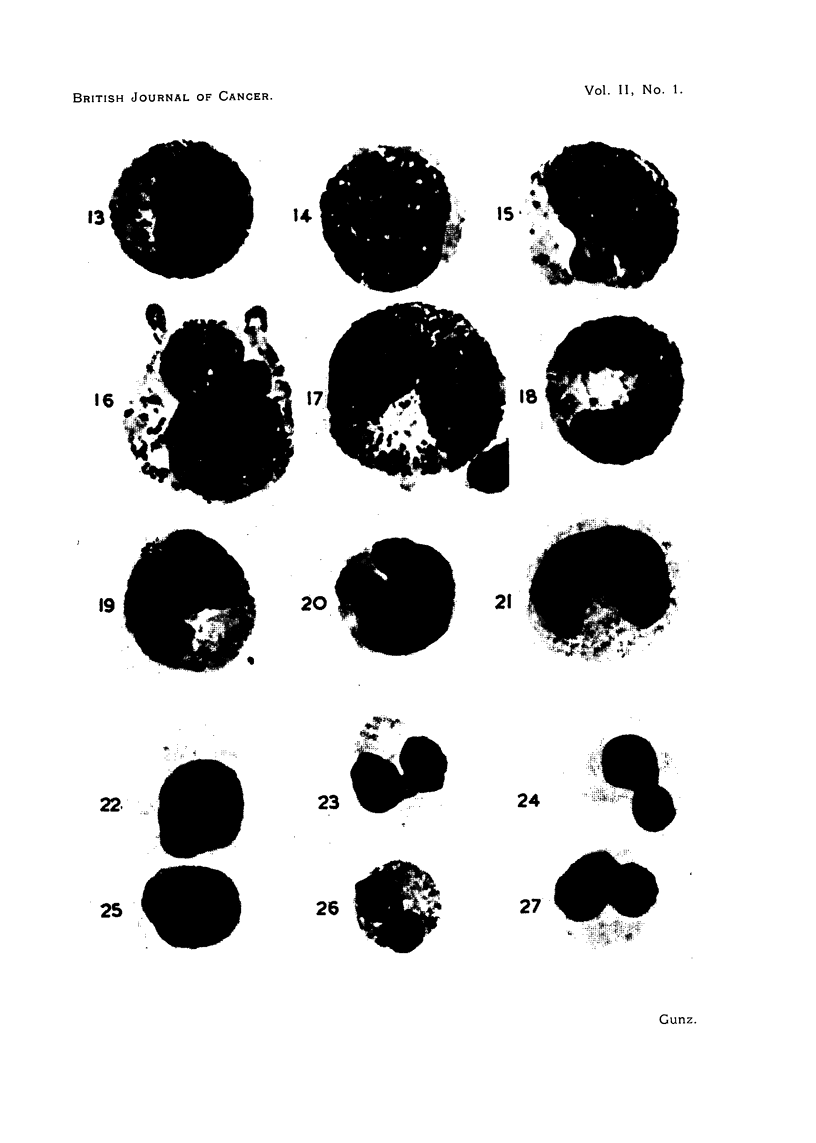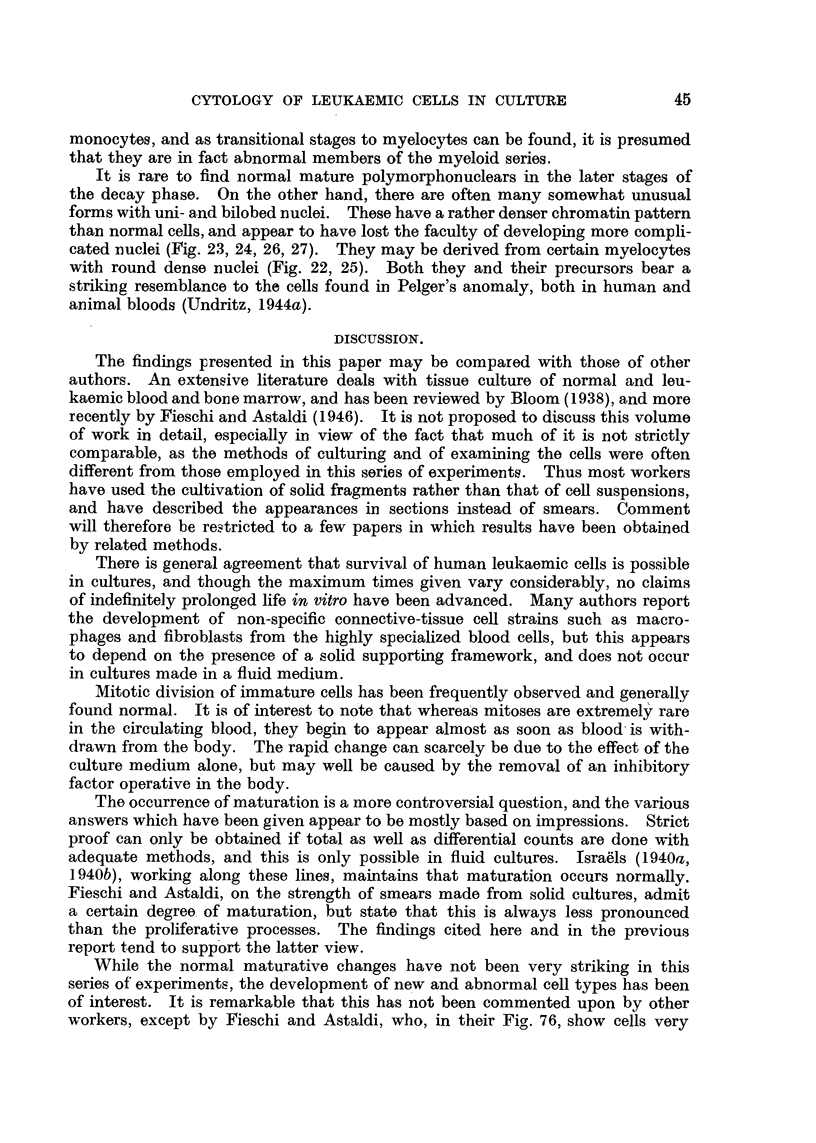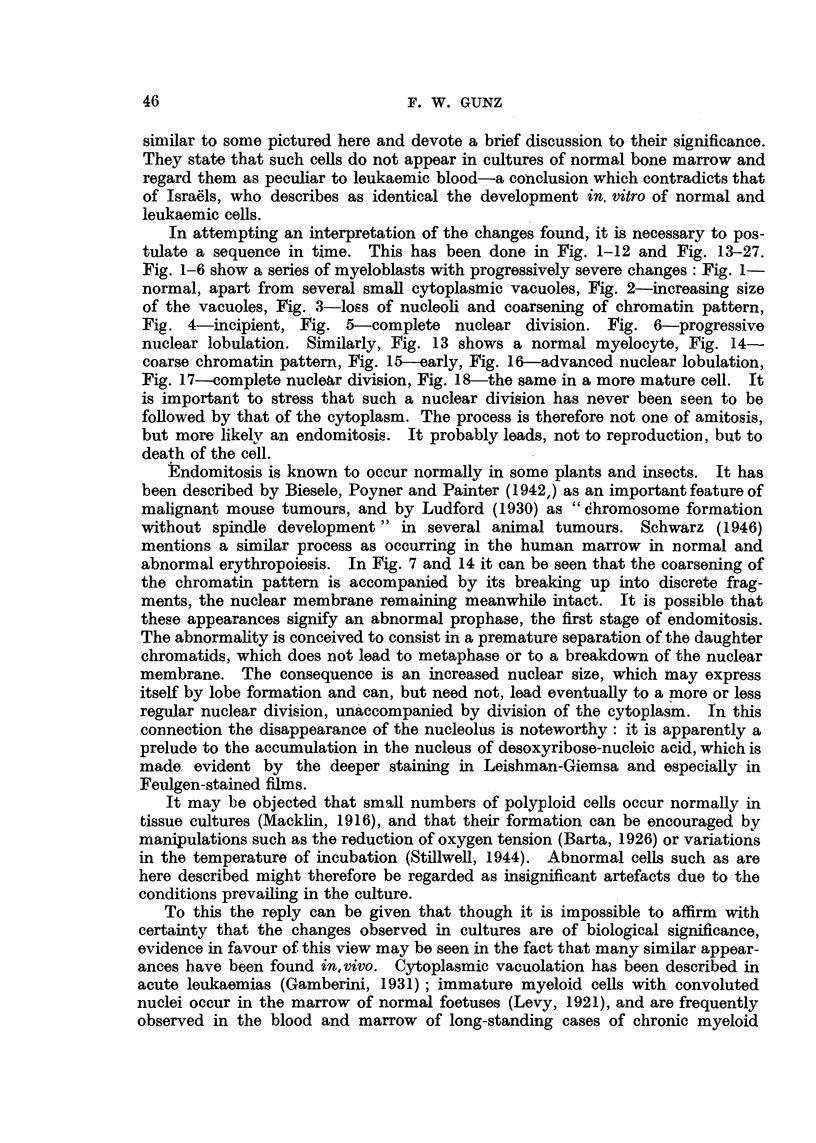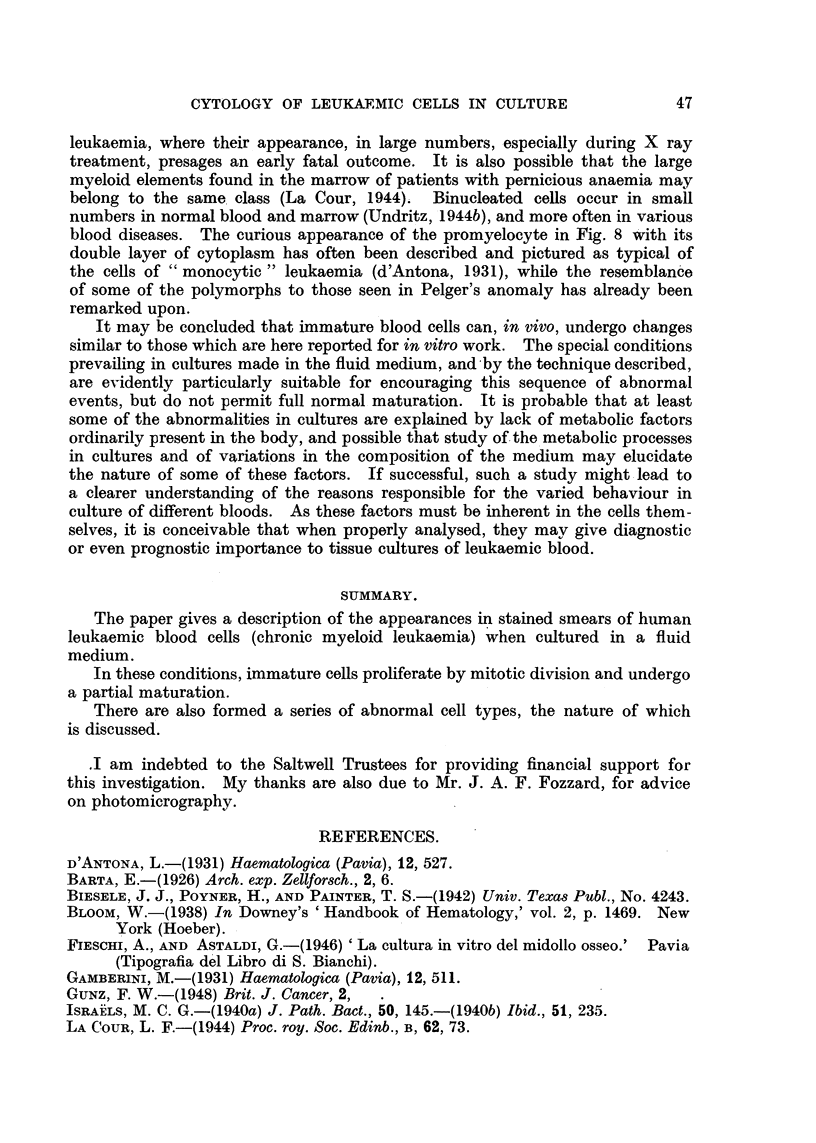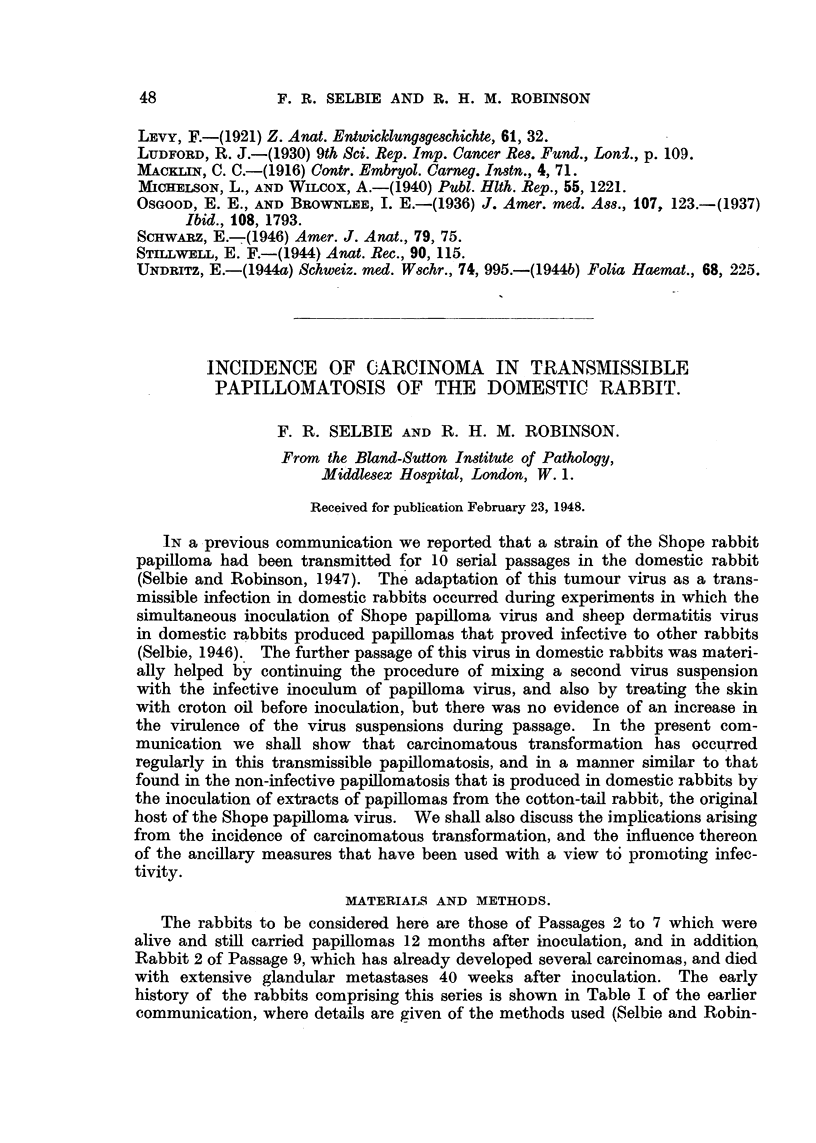# Culture of Human Leukaemic Blood Cells In Vitro. Normal and Abnormal Cell Division and Maturation

**DOI:** 10.1038/bjc.1948.6

**Published:** 1948-03

**Authors:** F. W. Gunz

## Abstract

**Images:**


FIESCHI, A., AND ASTALDI, G.—(1946) 'La Cultura in Vitro del Midollo Osseo.' Pavia (Tipografia del Libro).

FLECK, L., AND MURCZYNSKA, S.—(1947) *Texas Rep. Biol. Med.*, **5**, 156.

GEY, G. O., GEY, M. K., INUI, F., AND VEDDER, H.—(1945) *Gibson Island Conf. Monograph*, p. 321.

ISRÄELS, M. C. G.—(1940*a*) *J. Path Bact.*, **50**, 145.—(1940*b*) *Ibid.*, **51**, 235.

MOESCHLIN, S.—(1947) *Helv. med. Acta*, **14**, 279.

OSGOOD, E. E.—(1937) *J. Lab. clin. Med.*, **24**, 954.—(1939) *J. Amer. med. Ass.*, **109**, 933.

*Idem* AND BROWNLEE, I. E.—(1936) *Ibid.*, **107**, 123.—(1937) *Ibid.*, **108**, 1793.

PIERCE, M.—(1932) *Arch. Path.*, **14**, 295.—(1942) *Ibid.*, **34**, 538.

SCHREK, R.—(1946*a*) *Radiology*, **46**, 395.—(1946*b*) *J. cell. comp. Physiol.*, **28**, 277.

WALLBACH, G.—(1936) *Arch. exp. Zellforsch.*, **18**, 315.

WAYMOUTH, C.—(1947) *Proc. Soc. exp. Biol. N.Y.*, **64**, 25.

---

### CULTURE OF HUMAN LEUKAEMIC BLOOD CELLS *IN VITRO*. NORMAL AND ABNORMAL CELL DIVISION AND MATURATION.

F. W. GUNZ.*

*From the Department of Radiotherapeutics, University of Cambridge.*

Received for publication March 19, 1948.

THIS paper describes the fate of human leukaemic blood cells in an artificial fluid culture medium. A previous paper (Gunz, 1948) has dealt with the numerical aspect of the problem, outlining the increase and decrease of the counts of mature and immature cells from which can be traced the growth curve of the cultures, and discussing the factors which influence its shape. It is now proposed to follow in detail the changing appearances of the various cell types, as seen in stained films, and to attempt an interpretation of some of the cytological findings. The account will be confined to cultures of blood from patients with chronic myeloid leukaemia, as not enough cultures of other types of leukaemia have yet been available to permit a general description.

* Saltwell Research Student, Royal College of Physicians of London.

### TECHNIQUE.

A full account of the technique has been given in the above-mentioned paper, to which reference for details should be made. Briefly the method of culture is a modification of that first suggested by Osgood and Brownlee (1936, 1937), and consists of the separation of the leucocytes of heparinized venous blood from the red cells and plasma, of their suspension in a culture medium made up of equal parts of dried and reconstituted unfiltered adult human plasma and Ringer solution at pH 7·3, and of the distribution of the mixture into a series of culture tubes, the whole process being carried out by a " closed " technique with sterile hypodermic syringes and needles. In certain conditions the addition of embryo extract (E.E.) to the medium is desirable, as it has been shown that the number of mitoses in immature cells is thereby greatly increased. The cultures are inspected and counted at suitable intervals, and individual tubes are discarded after one count in triplicate. The medium is left unchanged throughout each experiment.

Films of concentrated cell suspensions made after gentle centrifugation were in the present series stained by a combined Leishman-Giemsa stain, and by Feulgen's method. The former, for which the formula was kindly supplied by Dr. M. W. A. Gatman,* was used because, when suitably buffered (pH 6·81–6·85), it gave more uniform results than the usual Romanowsky stains, without, at the same time, giving rise to any unaccustomed appearances in the cells. The Feulgen-stained smears were always confirmatory of the others, and will not be separately described. Differential counts were done on 500 leucocytes, and mitotic counts on 1000 immature cells from each smear.

### RESULTS.

This report is based on the findings in 31 cultures of blood from eleven patients with chronic myeloid leukaemia. Some of the patients were untreated, while others had had various forms of therapy. About 500–600 Leishman-Giemsa-stained films were studied in the course of these experiments.

The appearances described are those found in the basic culture medium. The addition of 5 per cent rat E.E. causes few differences, apart from raising the number of mitoses. The only divergent feature is the appearance of increased numbers of degenerate polymorphonuclears, probably owing to the metabolic stimulant action of the E.E.

From the cytological point of view, the life of each culture of leukaemic blood may be divided into two phases. The first, during which there is evidence of multiplication, and to a less extent of maturation, of the immature cells, reaches a climax during the first day, and comes to an end some time during the second 24 hours. The second phase is characterized by degenerative changes and death of all cell types, and lasts for a variable period from the second day of culture onwards. Death of the last cells occurs usually between the sixth and ninth days of culture, although in some instances it is delayed for several more days. Cultures differ from each other considerably in the rate and intensity of

* This is a modification of the Wright-Giemsa stain (Michelson and Wilcox, 1940), Leishman being used in the place of Wright powder. For staining, fix air-dried films in the concentrated stain for 1 minute: then dilute stain 1 : 10 with buffered water and leave for 10 minutes.

the various changes, and some of the factors responsible for this variability are by no means clear. Individual tubes of the same culture are, however, similar enough to each other to permit the drawing of a growth curve characteristic for them all.

I. *Growth phase.*

The name of this phase has been chosen, because during the first 36–48 hours of culture there is evidence of proliferation of the immature cells, provided by the presence of cells in mitotic division. From this, it does not follow that there is an increase in the total cell count of the cultures, for there is also a concomitant dying-off of mature cells. If, however, the mitotic index is high enough, as in cultures containing E.E., there may be an increase in the absolute number of immature cells. This means that proliferation outstrips maturation.

Mitotic figures, very rare in the peripheral blood, are regularly found in cultures from the second hour onwards. They occur mainly in myelocytes, but are also seen in myeloblasts. All stages of mitosis are frequently met with. Differential counts have been done as a routine, but the total numbers of mitoses are too small to permit precise conclusions on the phase distribution ; further, owing to the technique necessary in making smears, the recognition of telophases is often difficult, and their number probably greater than is apparent in counts. No gross deviation from the ordinary phase distribution has, however, been found. In particular, there is no evidence of a metaphase arrest in untreated cultures. The mitotic index reaches a maximum of four to six mitoses per 1000 immature cells at about the eighth hour of culture in basic medium, or of up to 19 per 1000 immature cells at about 24 hours, if E.E. is added.

The great majority of mitoses is normal in the first stage of the growth phase. Precise chromosome counts have not been possible, but there has been no single instance of a gross deviation from the diploid number. Minor abnormalities have occasionally been seen. Fig. 11 shows a myelocyte in anaphase, with one certain and another probable chromosome bridge. Fig. 12 shows an early myelocyte anaphase with several " lagging " chromosomes. It is interesting that these abnormalities which are commonly found following the application of X rays occurred in this instance in the absence of treatment. It is, however, usually impossible to decide whether the anaphase bridges are due to abnormal chromosome stickiness or to chromosome interchanges. In the later stages of the growth phase it is common to find pyknotic mitoses. These also occur occasionally in the decay phase.

It is difficult to determine how many immature cells can mature in cultures. Signs of limited maturation can often be observed during the growth phase. Thus, after 8 or 24 hours, one may find myeloblasts with a minimum number of cytoplasmic azurophil granules, a transition stage in the life of the cell at which there is probably no division. One can also often discover collections of normal juvenile polymorphonuclears, another shor -lived stage between immature and fully mature cells. Sometimes it is possible to demonstrate a rise in the absolute number of polymorphonuclears present. There is, however, no proof that a given cell in culture will undergo the complete cycle of normal changes from the myeloblast to the polymorphonuclear, and it is indeed unlikely that it can do so ; but as the growth phase continues, more and more cells show signs of abortive or abnormal maturation.

While there are comparatively few abnormalities of mitotic cells, abnormal "resting" cells can be found even in the earliest stages of culture. Their number increases during the later hours of the growth phase, and becomes very large during the decay phase. All types of myeloid cells are affected; abnormal immature cells may attempt to ripen, and more mature cells may themselves undergo an atypical development. Either the nucleus or the cytoplasm of a cell may be involved; and there is frequent asynchronism between the development of these two cellular constituents.

If an 8–24 hour culture is examined, the majority of cells will be normal, but there will also be an increasing proportion of immature cells showing one or more of the following abnormalities:—*Nucleus*: Chromatin pattern of myeloblasts and early myelocytes uniformly dense and coarse, resembling that of early erythroblasts (Fig. 7, 14); loss of the nucleoli of the earliest cells (Fig. 3); increase in nuclear size (Fig. 2); formation of nuclear lobes (Fig. 4, 15). *Cytoplasm*: Appearance and multiplication of vacuoles in myeloblasts and early myelocytes (Fig. 1, 2, 3, 4, 6, 7); separation of the cytoplasm of early myelocytes into two layers; an inner layer surrounding the nucleus and containing granules, and an outer clear basophilic one (Fig. 8, 9); appearance of pseudopodia, especially in very early cells (Fig. 2, 3, 4, 5, 8, 9, 10, 16, 17).

At 48 hours the changes are more advanced. The nuclear size has further increased and the lobes can be complex (Fig. 6, 16); other nuclei have divided into two parts, each of which is often the size of the parent nucleus (Fig. 5, 17, 18). Metamyelocytes with much convoluted or double nuclei also appear (Fig. 19, 20), and there are a number of completely pyknotic or smeared cells. The overall size of cells may, but need not increase in step with that of their nuclei.

Some changes also take place in the polymorphonuclears. There is an increasing number of multilobed cells, and at an early stage many of them become pyknotic. Early in the culture some juvenile forms appear, as mentioned above, and these seem to be able to age in the normal way.

II. *Decay phase.*

During this phase few mitoses are found, though occasional normal ones have been seen as late as the ninth and tenth days of culture. They cannot, however, influence the downward trend of the growth curve which, in this phase, decreases exponentially with time. The appearance of many of the "resting" cells is now very abnormal, and it is often difficult to classify them (Fig. 10, ? "haemohistioblast"). Monocytoid cells (Fig. 21) are frequently seen; as these occur in large numbers in cultures which were initially practically free from

---

EXPLANATION OF PLATES.

All figures are photomicrographs of abnormal cells from cultures of human blood (chronic myeloid leukaemia), two hours to five days old. The cells were stained in Leishman-Giemsa mixture at pH 6·81–6·85. Magnification × 2130, except Fig. 11, which is × 2600.

FIG. 1–7.—Abnormal myeloblasts from 2–48-hour cultures.
FIG. 8, 9.—Promyelocytes, 48 hours.
FIG. 10.—Unclassified primitive cell, 48 hours.
FIG. 11.—Myelocyte in anaphase of mitosis, showing double chromosome bridge, 24 hours.
FIG. 12.—Myelocyte in early anaphase, 48 hours.
FIG. 13–21.—Myelocytes in various stages of abnormal development, 2 hours–5 days.
FIG. 22, 25.—" Pelger " type of myelocytes, 5 days.
FIG. 23, 24, 26, 27.—" Pelger " type of polymorphonuclears, 48 hours–5 days.

Gunz.

Gunz.

monocytes, and as transitional stages to myelocytes can be found, it is presumed that they are in fact abnormal members of the myeloid series.

It is rare to find normal mature polymorphonuclears in the later stages of the decay phase. On the other hand, there are often many somewhat unusual forms with uni- and bilobed nuclei. These have a rather denser chromatin pattern than normal cells, and appear to have lost the faculty of developing more complicated nuclei (Fig. 23, 24, 26, 27). They may be derived from certain myelocytes with round dense nuclei (Fig. 22, 25). Both they and their precursors bear a striking resemblance to the cells found in Pelger's anomaly, both in human and animal bloods (Undritz, 1944*a*).

### DISCUSSION.

The findings presented in this paper may be compared with those of other authors. An extensive literature deals with tissue culture of normal and leukaemic blood and bone marrow, and has been reviewed by Bloom (1938), and more recently by Fieschi and Astaldi (1946). It is not proposed to discuss this volume of work in detail, especially in view of the fact that much of it is not strictly comparable, as the methods of culturing and of examining the cells were often different from those employed in this series of experiments. Thus most workers have used the cultivation of solid fragments rather than that of cell suspensions, and have described the appearances in sections instead of smears. Comment will therefore be restricted to a few papers in which results have been obtained by related methods.

There is general agreement that survival of human leukaemic cells is possible in cultures, and though the maximum times given vary considerably, no claims of indefinitely prolonged life *in vitro* have been advanced. Many authors report the development of non-specific connective-tissue cell strains such as macrophages and fibroblasts from the highly specialized blood cells, but this appears to depend on the presence of a solid supporting framework, and does not occur in cultures made in a fluid medium.

Mitotic division of immature cells has been frequently observed and generally found normal. It is of interest to note that whereas mitoses are extremely rare in the circulating blood, they begin to appear almost as soon as blood is withdrawn from the body. The rapid change can scarcely be due to the effect of the culture medium alone, but may well be caused by the removal of an inhibitory factor operative in the body.

The occurrence of maturation is a more controversial question, and the various answers which have been given appear to be mostly based on impressions. Strict proof can only be obtained if total as well as differential counts are done with adequate methods, and this is only possible in fluid cultures. Israëls (1940*a*, 1940*b*), working along these lines, maintains that maturation occurs normally. Fieschi and Astaldi, on the strength of smears made from solid cultures, admit a certain degree of maturation, but state that this is always less pronounced than the proliferative processes. The findings cited here and in the previous report tend to support the latter view.

While the normal maturative changes have not been very striking in this series of experiments, the development of new and abnormal cell types has been of interest. It is remarkable that this has not been commented upon by other workers, except by Fieschi and Astaldi, who, in their Fig. 76, show cells very similar to some pictured here and devote a brief discussion to their significance. They state that such cells do not appear in cultures of normal bone marrow and regard them as peculiar to leukaemic blood—a conclusion which contradicts that of Israëls, who describes as identical the development *in. vitro* of normal and leukaemic cells.

In attempting an interpretation of the changes found, it is necessary to postulate a sequence in time. This has been done in Fig. 1–12 and Fig. 13–27. Fig. 1–6 show a series of myeloblasts with progressively severe changes : Fig. 1—normal, apart from several small cytoplasmic vacuoles, Fig. 2—increasing size of the vacuoles, Fig. 3—loss of nucleoli and coarsening of chromatin pattern, Fig. 4—incipient, Fig. 5—complete nuclear division. Fig. 6—progressive nuclear lobulation. Similarly, Fig. 13 shows a normal myelocyte, Fig. 14—coarse chromatin pattern, Fig. 15—early, Fig. 16—advanced nuclear lobulation, Fig. 17—complete nuclear division, Fig. 18—the same in a more mature cell. It is important to stress that such a nuclear division has never been seen to be followed by that of the cytoplasm. The process is therefore not one of amitosis, but more likely an endomitosis. It probably leads, not to reproduction, but to death of the cell.

Endomitosis is known to occur normally in some plants and insects. It has been described by Biesele, Poyner and Painter (1942,) as an important feature of malignant mouse tumours, and by Ludford (1930) as " chromosome formation without spindle development " in several animal tumours. Schwarz (1946) mentions a similar process as occurring in the human marrow in normal and abnormal erythropoiesis. In Fig. 7 and 14 it can be seen that the coarsening of the chromatin pattern is accompanied by its breaking up into discrete fragments, the nuclear membrane remaining meanwhile intact. It is possible that these appearances signify an abnormal prophase, the first stage of endomitosis. The abnormality is conceived to consist in a premature separation of the daughter chromatids, which does not lead to metaphase or to a breakdown of the nuclear membrane. The consequence is an increased nuclear size, which may express itself by lobe formation and can, but need not, lead eventually to a more or less regular nuclear division, unaccompanied by division of the cytoplasm. In this connection the disappearance of the nucleolus is noteworthy : it is apparently a prelude to the accumulation in the nucleus of desoxyribose-nucleic acid, which is made evident by the deeper staining in Leishman-Giemsa and especially in Feulgen-stained films.

It may be objected that small numbers of polyploid cells occur normally in tissue cultures (Macklin, 1916), and that their formation can be encouraged by manipulations such as the reduction of oxygen tension (Barta, 1926) or variations in the temperature of incubation (Stillwell, 1944). Abnormal cells such as are here described might therefore be regarded as insignificant artefacts due to the conditions prevailing in the culture.

To this the reply can be given that though it is impossible to affirm with certainty that the changes observed in cultures are of biological significance, evidence in favour of this view may be seen in the fact that many similar appearances have been found *in, vivo.* Cytoplasmic vacuolation has been described in acute leukaemias (Gamberini, 1931) ; immature myeloid cells with convoluted nuclei occur in the marrow of normal foetuses (Levy, 1921), and are frequently observed in the blood and marrow of long-standing cases of chronic myeloid leukaemia, where their appearance, in large numbers, especially during X ray treatment, presages an early fatal outcome. It is also possible that the large myeloid elements found in the marrow of patients with pernicious anaemia may belong to the same class (La Cour, 1944). Binucleated cells occur in small numbers in normal blood and marrow (Undritz, 1944*b*), and more often in various blood diseases. The curious appearance of the promyelocyte in Fig. 8 with its double layer of cytoplasm has often been described and pictured as typical of the cells of " monocytic " leukaemia (d'Antona, 1931), while the resemblance of some of the polymorphs to those seen in Pelger's anomaly has already been remarked upon.

It may be concluded that immature blood cells can, *in vivo*, undergo changes similar to those which are here reported for *in vitro* work. The special conditions prevailing in cultures made in the fluid medium, and by the technique described, are evidently particularly suitable for encouraging this sequence of abnormal events, but do not permit full normal maturation. It is probable that at least some of the abnormalities in cultures are explained by lack of metabolic factors ordinarily present in the body, and possible that study of the metabolic processes in cultures and of variations in the composition of the medium may elucidate the nature of some of these factors. If successful, such a study might lead to a clearer understanding of the reasons responsible for the varied behaviour in culture of different bloods. As these factors must be inherent in the cells themselves, it is conceivable that when properly analysed, they may give diagnostic or even prognostic importance to tissue cultures of leukaemic blood.

### SUMMARY.

The paper gives a description of the appearances in stained smears of human leukaemic blood cells (chronic myeloid leukaemia) when cultured in a fluid medium.

In these conditions, immature cells proliferate by mitotic division and undergo a partial maturation.

There are also formed a series of abnormal cell types, the nature of which is discussed.

.I am indebted to the Saltwell Trustees for providing financial support for this investigation. My thanks are also due to Mr. J. A. F. Fozzard, for advice on photomicrography.

### REFERENCES.

D'ANTONA, L.—(1931) *Haematologica (Pavia)*, **12**, 527.
BARTA, E.—(1926) *Arch. exp. Zellforsch.*, **2**, 6.
BIESELE, J. J., POYNER, H., AND PAINTER, T. S.—(1942) *Univ. Texas Publ.*, No. 4243.
BLOOM, W.—(1938) *In* Downey's ' Handbook of Hematology,' vol. 2, p. 1469. New York (Hoeber).
FIESCHI, A., AND ASTALDI, G.—(1946) ' La cultura in vitro del midollo osseo.' Pavia (Tipografia del Libro di S. Bianchi).
GAMBERINI, M.—(1931) *Haematologica (Pavia)*, **12**, 511.
GUNZ, F. W.—(1948) *Brit. J. Cancer*, **2**, .
ISRAËLS, M. C. G.—(1940*a*) *J. Path. Bact.*, **50**, 145.—(1940*b*) *Ibid.*, **51**, 235.
LA COUR, L. F.—(1944) *Proc. roy. Soc. Edinb.*, B, **62**, 73.

LEVY, F.—(1921) *Z. Anat. Entwicklungsgeschichte*, **61**, 32.
LUDFORD, R. J.—(1930) *9th Sci. Rep. Imp. Cancer Res. Fund., Lond.*, p. 109.
MACKLIN, C. C.—(1916) *Contr. Embryol. Carneg. Instn.*, **4**, 71.
MICHELSON, L., AND WILCOX, A.—(1940) *Publ. Hlth. Rep.*, **55**, 1221.
OSGOOD, E. E., AND BROWNLEE, I. E.—(1936) *J. Amer. med. Ass.*, **107**, 123.—(1937) *Ibid.*, **108**, 1793.
SCHWARZ, E.—(1946) *Amer. J. Anat.*, **79**, 75.
STILLWELL, E. F.—(1944) *Anat. Rec.*, **90**, 115.
UNDRITZ, E.—(1944*a*) *Schweiz. med. Wschr.*, **74**, 995.—(1944*b*) *Folia Haemat.*, **68**, 225.

---

### INCIDENCE OF CARCINOMA IN TRANSMISSIBLE PAPILLOMATOSIS OF THE DOMESTIC RABBIT.

F. R. SELBIE AND R. H. M. ROBINSON.